# Through the eyes of the patients: a qualitative study of diabetes patients’ experiences navigating the healthcare system

**DOI:** 10.3389/fendo.2025.1588192

**Published:** 2025-08-26

**Authors:** P. V. AshaRani, Kumarasan Roystonn, Madhumitha Ramu, Yeow Wee Brian Tan, Fiona Devi, M. Iskander Shah, Peizhi Wang, Sum Chee Fang, Tavintharan Subramaniam, Lee Eng Sing, Chong Siow Ann, Mythily Subramaniam

**Affiliations:** ^1^ Research Division, Institute of Mental Health, Singapore, Singapore; ^2^ Khoo Teck Puat Hospital, National Healthcare Group, Singapore, Singapore; ^3^ Hougang Polyclinic, National Healthcare Group Polyclinics, Singapore, Singapore; ^4^ Future Primary Care, Ministry of Health (MOH) Office for Healthcare Transformation, Singapore, Singapore; ^5^ Saw Swee Hock School of Public Health, National University of Singapore, Singapore, Singapore

**Keywords:** diabetes, patient journey, experience map, qualitative, self-care, pre-diagnosis, unmet needs

## Abstract

**Background:**

Experience maps of patient journeys offer valuable insights into efficient and cost-effective diabetes care that reflects the needs experienced by the patients. This study describes the diabetes patients’ experience navigating the healthcare system.

**Methods:**

A phenomenological approach was adopted together with purposive sampling to achieve the study aim. One to one in-depth interviews were conducted with participants who had Type 2 diabetes (with and without diabetes related complications). A deductive thematic analysis was adopted for the study with data sufficiency achieved at thirteen interviews.

**Findings:**

Themes and codes were organized under three main stages: pre-diagnosis (stage 1), diagnosis and treatment (stage 2), and living with diabetes (stage 3). Stage 1 included themes for awareness and choice of care provider for initial care, and showed few care gaps, while stage 2 identified several service gaps and unmet needs. The themes that emerged included acceptance and life-facing diabetes and diabetes disease encounters, with several codes captured under the theme. Stage 3 included a theme for diabetes self-care. The usefulness of apps, good communication by the care team and facilitators of self-care were also mentioned. The major unmet needs perceived by the participant were patient-centeredness and personalized care in primary care settings.

**Conclusion:**

While the current diabetes care system was efficacious, areas for improvement exist, and patients expressed a desire for more patient-centered and personalized care, particularly in primary care settings. These findings offer valuable insights into T2DM management and highlight potential areas for enhancing healthcare delivery.

## 1 Introduction

An estimated 537 million individuals aged 20–79 suffer from diabetes, and it is a leading cause of mortality and morbidity worldwide. This number is projected to increase to 643 million by 2045 (1). In Southeast Asia, the prevalence of diabetes is currently at 8.7%, which is estimated to rise to 11.3% by 2045 (1). Diabetes and its associated complications (nephropathy, retinopathy, neuropathy, peripheral vascular diseases, and ulcers) carry a heavy disease burden and adversely affect the quality of life and life expectancy of patients. In 2021, around 6.7 million deaths among those aged 20–79 was attributed to diabetes and its complications (1).

Diabetes carries pervasive economic ramifications through direct healthcare costs and indirect impacts on productivity and disability. Healthcare expenditure for diabetes increased by 315%, from USD 232 billion in 2007 to USD 966 billion in 2021 globally (1). Sun et al. (2), estimated the cost will rise to USD 1054 billion by 2045. Rising costs can delay treatment for those with low socioeconomic status, leading to more undiagnosed cases (1). Healthcare systems and policymakers could address this through proactive measures to increase awareness, promote screening, treatment adherence, and prevent complications. Patient-centric approaches promise to enhance care efficiency and accessibility, improving quality and satisfaction. Achieving these outcomes requires a deep understanding of patients’ experiences and challenges throughout their healthcare journey.

Patient journey maps are powerful visualization methods describing patients’ interactions with the healthcare system. These maps capture longitudinal, sequential touch points across the care continuum, from initial diagnosis to follow-up (3). By identifying critical nodes like unmet needs and challenges, experience maps highlight factors promoting treatment adherence and satisfaction, allowing stakeholders to improve care quality. This study constructed an experience map of individuals living with diabetes, capturing stages from pre-diagnosis to self-care management. The map included stages such as symptom awareness, screening, treatment decisions, diagnosis, adherence, and disease management (3, 4). The journey began with symptom awareness and help-seeking, followed by interactions with healthcare providers, follow-up visits, and self-management.

Singapore, a Southeast Asian Island nation of 5.7 million, has a hybrid healthcare system with public and private facilities. The landscape includes over 2000 General Practitioner (GP) clinics, 26 polyclinics, 11 public hospitals, 10 national specialty centers, 9 private hospitals, and various other care facilities (5). The Ministry of Health has initiated community health centers, family medicine clinics, and primary care networks to enhance chronic disease management (6). GP clinics handle about 80% of primary care needs, while polyclinics offer preventive healthcare, education, and subsidized care. Singapore’s integrated public-private healthcare system and universal coverage with multiple affordability support layers offers unique experiences to the users that require synthesis of a journey map specific for this system. Our map aims to elucidate diabetes management challenges and facilitators, aligning with the ‘War on Diabetes’ campaign (7), which seeks to empower patients and healthcare systems for early detection, treatment, and improved quality of life for individuals with diabetes.

Previous studies have focused on service blueprints or customer journey maps from a system-level perspective (8, 9). This study employs experience maps capturing end-to-end experiences of individuals with diabetes chronologically, regardless of their transition between various care providers. Sali Davis (10) documented a patient-generated journey map describing her experiences throughout the diabetes journey, however, this approach is limited due to subjective bias and a narrow perspective, which affects the reliability and generalizability. Costa et al. (11), used a grounded theory approach to construct a “journey toward engagement in self-management” for diabetes foot ulcer that included five different phases: illness perception, symptom recognition, severity awareness, self-management education, and active engagement in self-care. However, there’s a scarcity of literature examining the entire diabetes journey from symptom awareness to ongoing management and follow up care. This study addresses this gap by mapping experiences of diabetes patients navigating the healthcare systems. The experience map will shed light on the met and unmet needs, emphasizing services requiring optimization. The study aims to visualize patients’ journeys through the healthcare system, exploring experiences and needs across various stages.

## 2 Methods

### 2.1 Design

The current study employed a phenomenological approach to capture the lived experiences of patients with diabetes as they navigated the healthcare system for their diabetes care. The study was reported as per Consolidated criteria for reporting qualitative research (COREQ) checklist (**Supplementary Table S1**).

### 2.2 Participants

Participants who were 21 years or older, citizens or permanent residents of Singapore, and proficient in one of the four official languages (English, Malay, Chinese, or Tamil) with a diagnosis of Type 2 diabetes (T2DM) were recruited (through referral by attending clinicians) from polyclinics and community-level Integrated Care Centers (ICCs). While polyclinics are part of the primary care system offering multidisciplinary holistic healthcare services, ICCs provide outpatient services and have Diabetes Centers providing community-level diabetes care with multidisciplinary care services housed under one roof. Exclusion criteria included individuals with Type 1 diabetes, those with cognitive impairments (as assessed by a clinician), non-residents, or those who were unable to speak any of the specified official languages. A maximum variation sample was ensured by recruiting those representing various age groups (21–35 years, above 35 years), sex, ethnicity (Chinese, Malay, Indian, and other ethnicities), and languages spoken (English, Chinese, Malay, and Tamil). We also included patients across different points on the disease trajectory (from newly diagnosed to those living with T2DM for many years), and diverse socioeconomic status (SES, as assessed by the medical social worker). The sample achieved good variation across healthcare clusters in terms of ethnicity, gender, socioeconomic status, and disease duration. However, younger participants were predominantly recruited from polyclinics. Written informed consent was obtained from all participants, and the study adhered to approved procedures from the National Healthcare Group Domain Specific Review Board (DSRB Ref: 2022/00339; approved on 1 February 2023).

### 2.3 Sampling and sample size

A purposive sampling was used to capture the holistic picture of the patient journey. Participants with a diagnosis of diabetes (Type 2 diabetes with and without complications) who had contact with the healthcare system were recruited. Code saturation (no new codes emerging from the data) was observed at 10 interviews and meaning saturation for all codes (indicated by richness, dimensions and depth of the data, absence of any new insights or nuances in the data and gaining a clear understanding of the relationship between the codes) was achieved at 13, as per the framework indicated by Hennink et al., 2017 (12).

### 2.4 Data collection

One-to-one, in-depth, semi-structured interviews were conducted either face-to-face (at home or in the meeting rooms in clinic) or through an online videoconferencing platform. Each interview lasted for 1 to 1.5 hours. The interview guide was derived through literature reviews and discussion with clinicians and stakeholder groups and was subsequently modified to accommodate recommendations from the care team, who considered clinic workflow, services available, and potential touch points. The interviewers introduced themselves to the participants (name, title, organization), explained the study’s purpose, and outlined procedures before commencing interviews. No prior relationship existed between the research team and participants. Given the team’s extensive experience in qualitative research, establishing rapport was prioritized before beginning the semi-structured interviews. Each interview started with general conversation about the participant’s background, daily activities, and interests, creating a comfortable environment for sharing experiences. Once rapport was established, the interview proceeded with questions focused on their diabetes journey. None of the interviews were repeated. All the interviews were audiotaped. The interview guide and details of the interviewers are included in **Supplementary Section 1**; **Table S2** and **S3**.

### 2.5 Reflexivity

The interviews were conducted by researchers experienced in qualitative methodology. Interviewer ethnicity was matched with participants where possible to better understand cultural contexts. A multidisciplinary team with biomedical sciences and psychology backgrounds conducted interviews for a broad perspective. While interviewers weren’t involved in diabetes care, some had family members with diabetes, aiding in understanding participants’ narratives without introducing bias. All the interviews were conducted by a main interviewer together with a note-taker, who also monitored the progress of the interview and ensured diversity in perspective while ensuring no bias was introduced during the interview. All interviewers (AR, MY, FD, KR, MR, IS, WP, and BT) were trained to be empathetic, nonjudgmental, and non-directive. Regular debriefing sessions were held to discuss the field notes, track progress, to identify methodological shortcomings, and for analytic considerations. While none of the researchers themselves had T2DM, several had close family members who had been diagnosed with the condition. Throughout the research process, the interviewers were aware of their assumptions and preconceptions and discussed it with the team to approach the data with openness and reflexivity. These steps prevented any personal experiences from introducing bias into data collection, analysis, or interpretation.

### 2.6 Data triangulation

The study employed methodological triangulation, investigator triangulation and data triangulation. Theoretical triangulation was not applicable in the current study which involved a realistic perspective. Data triangulation was achieved through maximum variation sampling, that ensured representation across demographic characteristics (age groups 21–35 years and above 35 years, gender, ethnicity - Chinese, Malay, Indian, and others), linguistic diversity (English, Chinese, Malay, and Tamil), and socioeconomic backgrounds. Additionally, participants were at different stages of their disease trajectory from recent diagnosis to those with long-term management across different primary care and integrated care settings. Methodological triangulation was accomplished through diverse data collection methods, including face-to-face and online videoconferencing interviews, supplemented by field notes and regular debriefing sessions. Investigator triangulation was implemented through ethnically matched interviewer-participant pairs where possible, and a dual interviewer approach (primary interviewer and note-taker) from multidisciplinary backgrounds.

### 2.7 Analysis

All the interviews were transcribed verbatim, and non- English language interviews were translated to English by experienced study team members or professional translation firms. All the transcripts underwent a quality check by another team member who was a native speaker of the language. The team convened at the end of the 3rd, 5th, and 6th interviews to discuss the field data and the preliminary codes from the interviews and to decide on any alternative strategies to improve the richness of the data. A deductive coding approach was used, guided by the literature (13, 14), to derive the overarching themes. The stages of the patient journey were broadly divided into pre-diagnosis, diagnosis and treatment, and post-diagnosis (living with diabetes). Journey stages were identified through literature searches, which included symptom awareness, treatment considerations, seeking care, treatment, and disease management (3, 4). The coding framework was developed through an iterative process. Six transcripts were distributed among the team members (AR, MY, FD, KR, MR, IS, BT, and WP) to familiarize and immerse themselves with the contents and to draw out preliminary codes that could be segregated under the overarching themes to create a coding framework. This framework was then piloted on two transcripts, leading to refinements in code definitions and hierarchies. The team met regularly to revise and discuss any discrepancies in codes and themes. The final codes and themes were combined, refined or discarded and a code book was generated using the final codes agreed upon by the team members. The code book included definitions, descriptions, inclusion and exclusion criteria, and examples of codes. The rest of the transcripts were screened for additional codes. The team approved the final coding framework before the final coding. Five interviewers did the final coding of the transcripts (MY, AR, MR, KR, and BT). The team employed a reflexive thematic analysis based on the data which was influenced by the reflective stance and ongoing self-reflection during the analysis phase. Coding discrepancies were resolved through regular meeting where the members reviewed the codes and section against their interpretations and reached a consensus as a team. The team achieved an inter-rater reliability of 0.72. All the analyses were conducted using Nvivo V.14 (QSR International).

## 3 Results

The sociodemographic characteristics of the sample are summarized in **Table 1**. Briefly, there were 4 males and 10 females, 5 participants from low SES and 2 from a younger age group (21–35 years). The sample showed a fair representation of various ethnic groups. Nine interviews were conducted in English, one in Chinese, and three in Malay. The duration of diabetes ranged from 1.8 years to 19 years, with 6 participants having 5 years or less and 7 living with diabetes for more than 5 years. Nine participants were on oral medications and 4 on insulin. All the participants on insulin were socio-economically disadvantaged. None of the participants were withdrawn from the study.

**Table 1:** Sociodemographic characteristics of the sample.

Sociodemographic characteristics	n
Ethnicity	
Chinese	7
Malay	4
Indian	2
Others	0
Sex	
Male	.4
Female	9
Marital status	
Single/never married	1
Married	7
Separated/widowed/divorced	5
Education	
Primary or below	3
Secondary school	3
Diploma	3
University degree and above	4
Employment status	
Employed	9
Unemployed	4
SES*	
High	8
Low	5
Personal income	
Below S$2,000	6
S$2,000- S$3,999	2
S$4,000 to S$5,999	1
S$6,000 to S$9,999	1
Above S$10, 000	3
Age	
Young (21–35 yrs.)	2
Others (>35 yrs.)	11
Duration of diabetes	
≤ 5 years	6
> 5 years	7
Medications	
Oral	9
Insulin	4

*SES, Socioeconomic Status.

**Figure 1:**
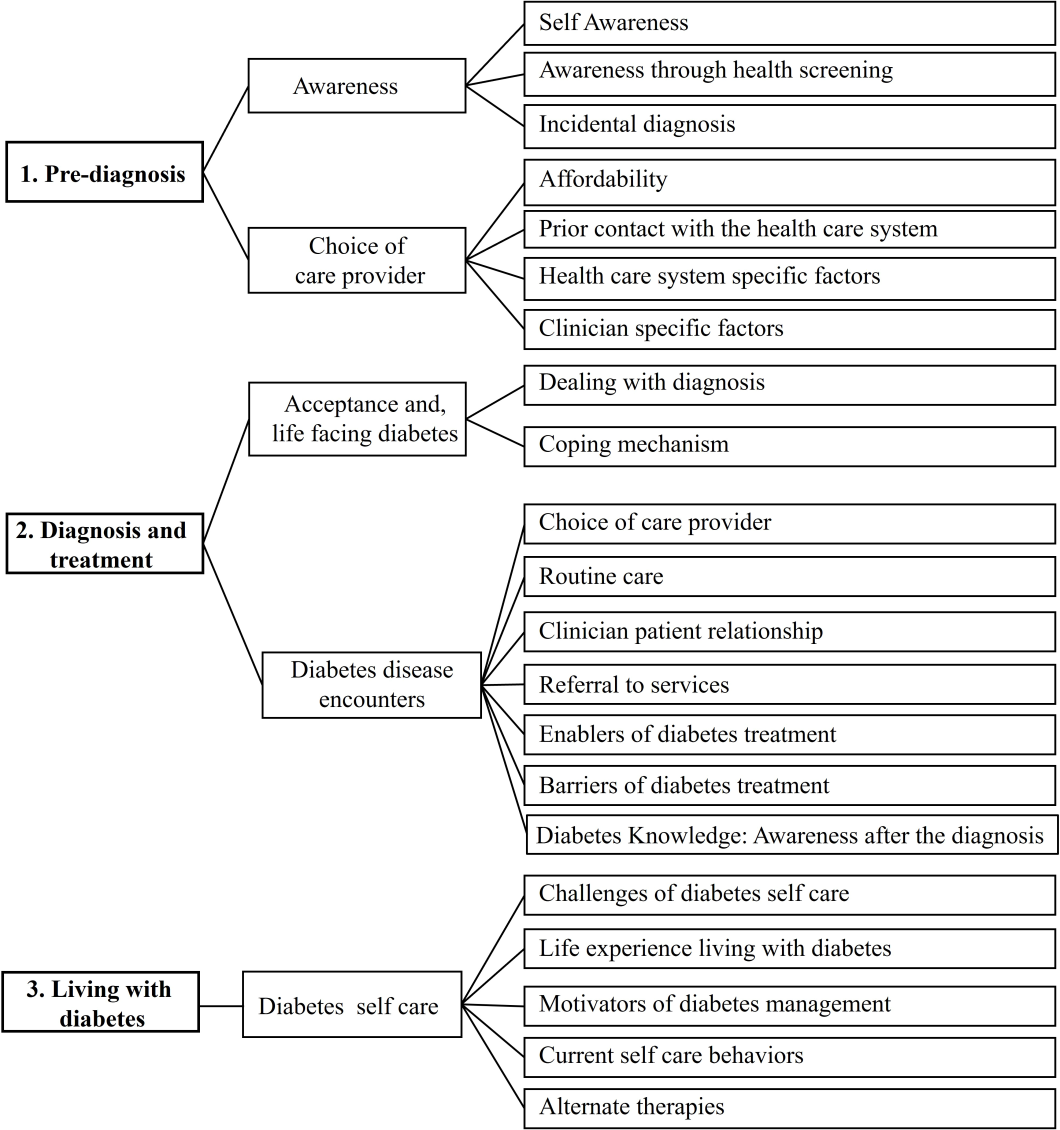
Major themes of the patient journey for diabetes patients.

Three main stages were included: pre-diagnosis (stage 1), diagnosis and treatment (stage 2), and living with diabetes (stage 3). The themes and codes are included in **Figure 1**.

### 3.1 Stage 1: pre-diagnosis stage

This stage delves into events in the pre-diagnosis period of diabetes.

#### 3.1.1 Awareness

##### 3.1.1.1 Self-awareness

Half the participants noticed diabetes symptoms and initiated primary care appointments, often due to known risk factors like family history. Those experiencing symptoms like frequent urination and excessive thirst related them to diabetes. Others sought information from family, friends, or search engines to identify potential causes. Participants who were caregivers for diabetic family members were vigilant about their higher risk and symptoms, prompting home glucose monitoring or self-initiated screening appointments. No significant treatment delays in diagnosis were indicated in their narratives.

“I didn’t realize that I have diabetes actually, because I didn’t have any symptoms. When my sister had it, only then, after that, I thought, I should go and have a check-up.(47 years, Female)

Overall, participants demonstrated varying patterns of help-seeking behaviors based on their SES. Those from higher SES generally showed proactive help-seeking behavior, influenced by symptom recognition, awareness through family history, and prior caregiving experience with diabetic relatives. Their narratives highlighted how government initiatives for regular health screening and early diabetes detection enhanced their self-awareness about diabetes risk. However, the majority of participants belonging to lower SES either did not experience symptoms or failed to recognize them, leading to diabetes-related complications and subsequent detection during emergency care. The remaining participants from this group, or their family members, were able to recognize the symptoms, which prompted timely help-seeking behaviors.

This interplay of personal experiences, socioeconomic factors, and public health awareness appeared to significantly influence the timing and pathway to diabetes diagnosis, with higher SES individuals generally achieving more timely diagnoses compared to those from a socio-economically disadvantaged background.

##### 3.1.1.2 Awareness through health screening

Over a quarter of participants attended regular health screenings, either self-initiated, for insurance, or as part of workplace annual health checks. These screenings revealed abnormal glucose results, prompting further help-seeking. Participants discussed results with friends or family, who encouraged additional diagnostic tests in primary care settings.

“I was diagnosed with diabetes, during a routine blood test, that I did as a health check that I did at the end of, I think 2021” (46 years, male)

Overall, multiple factors converged to facilitate early diabetes detection, particularly among those with from high SES. As evident in the narratives, government-subsidized screening programs, helped to promote awareness toward health screening among individuals, and supportive workplace health policies played crucial roles. These systemic enablers, combined with social support networks, encouraged help-seeking behaviors. Those from higher SES were clearly in a better position to leverage these resources and support systems, demonstrating better ability to access and utilize available healthcare services for early diabetes detection and management.

##### 3.1.1.3 Incidental diagnosis

Nearly a quarter of participants were asymptomatic, with hyperglycemia detected while seeking treatment for other health conditions like surgical procedures, urinary infections, or emergency care. This pattern was particularly prevalent among those those from lower SES. Diagnostic tests by attending clinicians revealed higher glucose levels, leading to further clinical examinations. The narratives highlighted an important pathway to diabetes diagnosis through incidental detection during treatment for other health conditions, especially among lower SES individuals who were less likely to engage in routine preventive healthcare. This finding underscores the critical role of healthcare providers in routine diabetes screening, particularly for those from lower SES who might otherwise remain undiagnosed until complications arise.

“I got some heartburn, or I can’t remember what. I didn’t feel well, then I had to go to A&E. That’s when I realized. He was saying that my blood sugar is very high, so he asked me to go and check”. (35 years, female)

#### 3.1.2 Choice of care provider

Almost all the primary contacts (initial contact point for diagnosis and care) for diabetes care were from primary care providers (GPs) or polyclinic doctors).

##### 3.1.2.1 Affordability

Participants chose initial contact points based on overall affordability of diabetes care in different settings. They were aware of long-term costs and compared consultation, medication, and potential complication-related expenses. A quarter chose polyclinics for affordability and subsidized care. Some working adults selected providers from their company’s panel list. Overall, the choice of healthcare provider was influenced mainly by financial considerations, with clear awareness of both immediate and long-term healthcare costs. The availability of subsidized care through polyclinics and workplace healthcare benefits emerged as important factors in their decision-making process for diabetes care.

“He (refers to specialist) suggested that why don’t I see a polyclinic for this instead of seeing a private GP. The difference in the cost of the medicine, the exact medicine is tremendous. “ (46 years, male)

##### 3.1.2.2 Prior contact with the healthcare system

Nearly half of participants or their families had been seeking treatment with their chosen care provider for other medical problems. They felt confident that the GP’s familiarity with their past and current medical history would facilitate holistic care. In general, the narratives showed that the participants valued the continuity of care and perceived that their established relationship with healthcare providers would contribute to a smooth treatment journey

“Before that, I’d been bringing my grandma, my late grandma to the polyclinic for like years. So like I’m familiar with the system, it’s easy to book, so that’s why I did it.” (27 years, female)

##### 3.1.2.3 Healthcare system specific factors

Nearly half of participants selected care providers based on convenience, ease of booking appointments, and shorter waiting times. They compared workflows of different healthcare settings, choosing those offering flexibility in navigating visits. Those referred by secondary or tertiary institutions had appointments automatically routed to the nearest polyclinic. The narratives highlighted how healthcare provider selection was strongly influenced by operational factors, particularly the flexibility in appointment systems and waiting time management.

“The visit is normally, if morning, if I go maybe, it’s a long queue already, the reception will tell me, “You come after maybe one or two hours later, or afternoon I’ll give you an appointment, the first one, or what.” Then, sometimes, I’ll go back. I don’t wait there. I don’t sit and wait. I’ll come back home. … I will go. 2:30 PM they will open again. So sometimes, I will be the first person to go and see him (refers toGP).” (47 years, female)

##### 3.1.2.4 Clinician specific factors

Participants considered clinician-specific factors like friendliness, knowledge, empathy, and medical skills when choosing providers. Their descriptions revealed a desire for long-term therapeutic relationships, recognizing diabetes as chronic. Selection of providers included personal characteristics of doctors, mostly observed for GPs whom they had visited earlier for minor ailments. The narratives revealed how participants carefully evaluated clinicians’ interpersonal qualities, professional competence, and personal experiences with diabetes when selecting providers, recognizing the importance of establishing meaningful therapeutic relationships for their long-term diabetes journey.

“He (refers to the GP) had diabetes himself, so there was a lot of empathy.” (46 years, male)

In summary, the narratives revealed distinct socioeconomic disparities in diabetes diagnosis pathways. Higher SES individuals demonstrated proactive help-seeking behaviors, benefiting from government health initiatives, workplace policies, and better symptom awareness. In contrast, majority of those from a lower SES often experienced delayed diagnoses, typically discovered through emergency care or incidental findings during treatment for other conditions. While systemic enablers and social support networks facilitated early detection, these benefits were predominantly accessed by those from higher SES. The findings highlight the critical need for targeted interventions to address these socioeconomic disparities in diabetes detection and care.

### 3.2 Stage 2: diabetes diagnosis and treatment

This stage included patients’ narratives on the journey through the actual diagnosis of diabetes and treatment phase.

#### 3.2.1 Acceptance and, life facing the diagnosis

##### 3.2.1.1 Dealing with the diagnosis

Participants had mixed responses to diagnosis, with most struggling to cope. Some of the participants were not emotionally affected, attributed to their diabetes knowledge. Some realized diabetes was not life-threatening if controlled well.

“There was nothing threatening or scary about it. I think it is a well-understood, uh, condition. Yeah. And entirely controllable. So-so there was nothing unpleasant about it.” (46 years, male)

For others, diagnosis was a shock, causing inner turmoil about the future journey. This emotional turmoil was particularly profound among younger participants, predominantly stemming from their preconceived notion that diabetes is an age-related condition and their apprehension about the life-long implications of managing a chronic disease. The complexity of their emotional responses was further compounded by uncertainties surrounding daily diabetes management and anticipated lifestyle modifications which was described as “mentally draining”. No notable differences in perspectives were observed across SES.

The narrative of a young participant highlighted how the diabetes diagnosis in early adulthood triggered profound emotional distress, stemming from feelings of isolation and the need for abrupt transition from a carefree life to managing a chronic condition. Their account emphasized how the current ‘cookie-cutter’ approach to post-diagnosis support failed to address younger patients’ unique psychological needs, highlighting the importance of age-appropriate emotional support and the challenge of processing an unexpected chronic disease diagnosis during young adulthood.

“Mentally, it wasn’t great, emotionally as well because I felt like I suddenly was being punished if I can put it that way. I had trouble accepting it because it was, like, uh, it’s a lifelong thing. That’s how it was framed to me. So, it felt a bit like, “Oh, your life is over.” That was the initial reaction that I had … it was very hard too, because I think there was no support.” (27 years, female)

Participants without family history or predisposing factors expressed denial upon diagnosis, largely attributable to their belief that genetic predisposition constitutes the primary risk factor for diabetes. Their narratives revealed a misconception that the absence of family history conferred protection against developing the condition, leading to a state of denial.

The narratives revealed a spectrum of emotional responses to diabetes diagnosis, from acceptance to distress. Prior knowledge and understanding of diabetes appeared to facilitate coping, while those without perceived risk factors experienced greater emotional challenges. These varied reactions highlighted how personal context, age and diabetes awareness influenced participants’ initial psychological adjustment to their diagnosis.

##### 3.2.1.2 Coping mechanism

Family, friends, and healthcare teams played valuable roles in helping patients cope with diagnosis. Older participants found the healthcare team supportive and guiding through transition. For younger participants, family discussions provided support to overcome stress, while friends with diabetes shared empathetic, motivating stories, helping them view it as an opportunity for positive change. Overall, the narratives showed how participants mobilized multiple sources of support to cope with their diabetes diagnosis, with different support systems resonating across age groups. Older participants primarily relied on guidance from healthcare team, while younger participants drew strength from family and peers.

“We found out that we both had the same condition, we started trading stories, like, “Oh, how did you deal with this? How did you deal with that? How did you feel, initially, during your diagnosis?” So, that changed things.” (27 years, Chinese)

#### 3.2.2 Diabetes disease encounters

##### 3.2.2.1 Choice of care provider for specialist care

This theme emerged from participants who shifted to specialist diabetes care. Over a quarter selected specialists independently, influenced by previous healthcare experiences, known clinicians, reluctance to visit a hospital, or knowledge of ICC for diabetes care. Some sought advice from family or friends with diabetes seeing specialists. Nearly half were referred to polyclinics, specialists, or ICC by GPs or panel doctors due to complications, perceived increased risk, or difficulty in achieving optimal blood sugar levels. Participants also considered cost, convenience, care quality, clinic hours, and waiting times when choosing specialists.

The narratives revealed complex decision-making processes in selecting specialist diabetes care providers that included previous healthcare experiences, recommendations from social networks, and perceptions of care settings - notably the ICC’s non-clinical, integrated environment which many participants described as a ‘serene sanctuary’. Healthcare system factors drove transitions through referrals, particularly when complications arose, with participants actively weighing these factors against their personal circumstances. The ICC emerged as a preferred choice across these dimensions, offering both holistic care and operational efficiency. This was particularly valued by participants from low SES backgrounds with complex care needs, who also cited long waiting times at primary care as a deterrent factor. The choice between polyclinics, ICC and GP clinics reflected individual trade-offs between cost, waiting times, complications, and consultation duration.

“So now, I think the government is also promoting the idea of seeing GPs, having a family doctor. I received that message, but I didn’t participate. I still stick with my clinic X (ICC). because I feel like I don’t want to queue and wait without an appointment with a GP. So, I’d rather go with clinic X (ICC) because I can get an appointment, so I didn’t participate. …” When I went there, my first impression was, “Wow, is this a hospital or a medical center?” It’s very convenient, and there’s a big food court on the second floor, so eating is very convenient.” (61 years, female)

##### 3.2.2.2 Routine care

This code captures participants’ routine diabetes clinic encounters, including experiences of treatment efficacy, affordability, follow-up care, and goal setting. Participants perceived improved symptoms and optimal blood glucose levels as treatment efficiency indicators. Their experiences highlighted an evolving treatment journey, involving optimal blood glucose levels, medication adjustments and dosing modifications, with dose reductions perceived as treatment success. Some participants, particularly those using Continuous Glucose Monitoring devices (CGM), described gaining deeper insights into the relationship between lifestyle factors and glucose control.

“So I went from, uh, three pills of 250 mg dose a day to two pills because, uh, I was controlling my diet and exercise, um, at the same time. So after s-several consults, um, over a period of maybe one and a half years, uh, my dosage was reduced to two pills a day.” (46 years, male)

Narratives revealed cost disparities among care providers for routine care. While CGMs were considered useful, high costs deterred many from using them, with use being mainly among those of higher SES. Metformin was affordable, but overall diabetes care costs were high due to daily dosing. Participants knew about regular follow-up needs which varied between 3 to 6 months depending on their needs and progress. Those with suboptimal glucose readings were given follow up more intensely.

Almost all had collaborative goal setting with clinicians, ranging from maintaining healthy HbA1C to complex goals like weight loss and strict glucose control, involving food diaries and daily finger prick test result logs. The narratives revealed age-related differences in perceptions of goal-setting approaches. Younger participants experienced weight management-focused goals and intensive lifestyle modifications as potentially stigmatizing and prescriptive, perceiving limited consideration of their individual needs and expectations. In contrast, older participants, while perceiving their treatment targets as challenging to attain, described a more collaborative approach with healthcare providers. They expressed appreciation for the healthcare team’s supportive guidance and perceived investment in their wellbeing.

“It’s just like this is your goalpost. Good luck. (27 years, female)

Participants’ narratives also revealed ICC’s personalized approach through examples of successful patient-provider negotiations, where clinicians balanced clinical goals with patient preferences, offering individualized solutions ranging from medication adjustments to dietary modifications while maintaining patient autonomy and comfort. Overall, the narratives revealed how routine diabetes care experiences varied across different patient groups - with age (younger participants finding weight-focused goals stigmatizing versus older participants experiencing collaborative care), socioeconomic status (low SES groups facing cost barriers while high SES groups accessed advanced monitoring), and care settings (ICC offering more personalized care compared to primary care and other public or restructured hospitals) emerging as key differentiating factors.

##### 3.2.2.3 Clinician patient relationship

Participants shared positive and negative aspects of their care team. Positive aspects included clear communication about condition and progress, personalized care, friendly interactions, consideration of patient preferences, instilling trust in prescribed medications, and lifestyle interventions based on daily activities.

The doctor (refers to ICC) will get for me an appointment, then she will call me. She will call me directly, my phone, and ask, “This is why you never come for the appointment.” She is very concerned about me. I thought wow that is really nice, “At least somebody is concerned about me, especially my Dr. X.” (53 years, female)

Negative aspects included not having the same doctor at polyclinic visits, communication problems, non-empathetic attitudes, lack of sincerity, and unrealistic goal setting. These experiences were reported across both primary care settings and public or restructured hospitals, particularly among those referred for further management of complications. Patients were disappointed with clinician rotation in polyclinics, perceiving a lack of rapport. Some felt polyclinic appointments were cursory and did not justify long waiting times. Communication barriers existed for those with limited English proficiency. Nearly half expressed concerns about rotating doctors and short consultations, likely due to mismatched expectations between patients desiring detailed discussions and doctors balancing consultation time with high patient volumes.

“He (refers to a clinician in a public hospital) would scold me when he saw my results not improving. He scolded me, saying, “Why did you come to see me? You can’t control it. People like you, even with this disease, can’t control it. If it were as easy as you say, no one would need injections.” So, I told the doctor, “You are also a diabetic patient because he was very thin. You are not losing weight, so are you saying thin people can’t have diabetes? … Actually, you should focus on how to treat me instead of scolding me.” (61 years, female)

Overall, the narratives showed the multifaceted nature of patient-provider interactions regardless of age group, where continuity of care and quality of communication emerged as key determinants of care experiences. The clinician-patient relationship was dichotomous: positive therapeutic relationship was characterized by personalized care and effective communication, while negative interactions stemmed from systemic constraints. These constraints, particularly evident in polyclinics, included provider rotation and time limitations, which affected therapeutic rapport and consultation quality. Communication barriers and divergent expectations regarding consultation depth emerged as significant challenges in establishing effective clinician-patient relationships. Majority of those from low socioeconomic background were receiving care at the ICC, with their narratives highlighting positive experiences that contrasted with their previous encounters with operational constraints in primary care, suggesting that referral patterns for complex care needs may have facilitated access to more inclusive specialist care.

##### 3.2.2.4 Referral to services

This code captured participants’ referrals to ancillary services and specialists, a necessary step in diabetes care. Nearly all utilized these services, with varying frequency based on diabetes severity and complications. All found the transition smooth, with efficient scheduling and reminder systems. Participants acknowledged this as a necessary step, one calling it a “standard operating procedure” for diabetes patients. Most had positive experiences with specialist referrals and care. Those following ICC were referred to specialists in the same building, which they found helpful and timesaving.

“Because she (the dietician) will tell me what to eat. Like before I can eat two bananas, say, “No, no, cannot, just eat one.” Once a day should be enough. Apple and you cannot eat apple, the big apple. So, just the small size of apple. So, all those-all those small, small things are, but actually it helps a lot.” (54 years, female)

The narratives revealed how participants viewed referrals to ancillary services and specialists as an integral component of their diabetes care journey, with efficient transitions and scheduling systems facilitating access to comprehensive care. Both primary care and ICC settings demonstrated smooth referral processes, though ICC participants particularly valued the convenience of co-located services.

##### 3.2.2.5 Enablers of diabetes treatment

The narratives revealed distinct experiences across care settings while highlighting system-wide efficiencies. Both primary care and ICC settings demonstrated strong operational enablers including efficient appointment systems, digital solutions (Health Hub app), medication delivery services, and integrated health records. However, notable differences emerged in care delivery experiences. Primary care settings, while praised for operational efficiency and digital solutions, faced challenges with crowding and time constraints, leading some participants to prefer telemedicine for longer consultations. In contrast, the ICC emerged as particularly favorable due to its integrated approach - offering shorter waiting times, same-day multiple specialist appointments, longer consultation durations, and enhanced facility amenities. The healthcare team experiences also differed: while both settings demonstrated instances of empathetic care, ICC participants consistently emphasized the personalized attention, thorough care approaches, and the comfortable, non-clinical atmosphere that facilitated regular follow-up. These narratives suggest that while both systems maintained operational efficiency, the ICC’s integrated model better addressed the holistic care needs of diabetes patients.

“That’s what I say the waiting times (at ICC) also is very short. Sometimes I think I going to have a checkup, I don’t know how long it will take like two or three hours but when I go there it is much faster than my expectation. When I go five minutes for the dropping, five minutes for the checkup (refers to blood routine anthropometric measures), five minutes for the X-ray. It’s just like I don’t have to wait so long. Next, even when I walk in already, they call my number or name. Then I was, “Wait. I’m coming” then I’ll go.” (47 years, female).

##### 3.2.2.6 Barriers to diabetes treatment

Barriers identified included clinician rotations, crowded polyclinics, short consultations, affordability, staff shortages, clinic hours, treatment quality, parking facilities, and multiple referrals. Long waiting times in polyclinics and hospitals were most common barriers cited, attributed to popularity of the clinic and large patient volumes. This made the diabetes journey difficult, with long queues for registration, tests, and consultation. Long waits were perceived to cause shorter consultations, causing dissatisfaction. Limited operating hours (8.30 to 5 p.m.) were a barrier for working adults who had to take medical leave for managing these appointments.

“I said, “Try to go there.” He (family member) said that the Polyclinic X has Saturday appointments, and he can only go on Saturdays because he works on weekdays. But ICC doesn’t have weekend appointments, only weekdays, so he can’t go either. So I said the government’s intention is actually good, but they didn’t consider people who are really working. Not everyone can take time off to follow up. Even though it’s every three or four months, it’s still a burden.”(61 years, female)

Healthcare team-related barriers included generic advice, lack of personalized care and patience, non-patient-centeredness, lack of empathy, unhelpful advice, disrespect, and poor communication. Some patients reported being scolded for uncontrolled glucose levels or admitting to sugary food consumption. A young participant felt stigmatized by unempathetic treatment, perceiving advice for drastic lifestyle changes with repetitive, weight-focused conversations during visits as punishment rather than care.

“Sometimes, the way they talk, it’s - we know we can’t afford it, but they could be more polite in their conversation. Sometimes, the conversation can be a bit harsh. or, when there are a lot of people around, it’s embarrassing, right?” (68 years, female)

The narratives revealed distinct barriers across healthcare settings and patient demographics. System-level barriers predominantly affected primary care settings, which included long waiting times, operating hours, and perception of lack of continued care due to inability to see the same doctor consistently. While ICC largely avoided these operational challenges (except operating hours), both settings faced affordability concerns which was less emphasized for primary care, particularly affecting patients from low SES group. Healthcare team-related barriers showed notable variations across age groups and care settings. Younger participants reported expectation mismatch and more care needs in terms of support groups and personalized programs. Communication barriers were more pronounced among older adults and those with limited English proficiency. Some participants expressed concerns about generic dietary advice and communication gaps, particularly in managing complex cases, suggesting a wider gap at the national level.

##### 3.2.2.7 Diabetes knowledge: awareness after diabetes diagnosis

This code captured the trajectory of gaining diabetes knowledge after the diagnosis phase. Participants recounted that CGM debunked myths about suitable foods for diabetes and helped them to choose food wisely. participants said that they started reading more about diabetes on the internet and through social media. Some found their disease journey itself informative. Others relied on friends, family, or search engines for diabetes information. Government campaigns, like Nutri-Grade labeling measures for sugary drinks, were cited as information sources. Participants gained knowledge from healthcare teams during their own care or while caring for family members with diabetes. Overall, common knowledge sources included search engines, YouTube, family, friends, and healthcare teams.

“The diabetes medicine, metformin, right, has an anti-aging effect. So, it is documented but usually not conveyed to the patient because, it is not meant to be prescribed as an aesthetic medication. Your-your wrinkles will disappear, the skin will tighten.” (47 years, male)

### 3.3 Stage 3: living with diabetes

This theme captured experiences in their daily lives, which included self-care habits, challenges and motivators in self-care, and alternative therapies they were adopting.

#### 3.3.1 Diabetes self-care

##### 3.3.1.1 Challenges of diabetes self-care

Factors identified included personal factors such as lack of self-discipline (giving up tasty food, disliking healthy foods, stress eating, alcohol consumption), limited resources, laziness, fear of medication side effects, forgetfulness (medication and appointments), financial and work-related barriers, and difficulty sustaining changes. Over a quarter found giving up tasty food challenging. Some admitted to indulging in comfort foods when stressed. Working adults struggled with diet control due to work related travel and office gatherings. Participants also cited the financial crisis as a reason for medication non-adherence and self-care neglect. Nearly half of the participants also felt that work and family commitments were self-care challenges for them.

“Sometimes it’s hard to, I guess sometimes it’s harder to control the diet if, like, let’s say I’m going through stress or like psychological yeah like stress from like work or personal life. So that is probably the hardest thing. because when I’m not feeling great then I’m more prone to indulging in things that I like or like alcohol, alcohol’s bad for me.” (27 years, female)

Overall, the narratives of younger participants described stress-related eating and work-social gathering challenges to diabetes self-care. Older participants expressed more concerns about medication complexity and forgetfulness. Work-life balance emerged as a significant barrier across age groups, though working adults faced unique challenges in maintaining self-care routines around professional commitments. While care setting did not notably influence self-care challenges, financial constraints particularly affected the ability of participants from low socioeconomic background to maintain consistent self-care practices, such as regular glucose monitoring and medication adherence.

##### 3.3.1.2 Life experience living with diabetes

This code captured the impact of diabetes on their daily lives. The participant shared how they changed their lifestyle or habits after the diagnosis as they prepared for the life ahead with diabetes. This included moderating or avoiding their favorite food that had high carbohydrates, drastic changes in lifestyle incorporating exercise and diet changes, having constant mood changes, feeling weak, having to live with complications and taking multiple medications, the need for assistance in self-care, and adjustments to work.

Overall, the narratives revealed diverse experiences of living with diabetes, with impacts varying across age groups and disease severity. Younger participants emphasized lifestyle modifications and workplace adjustments, particularly around managing long working hours and social activities. Older participants and those with complications reported more profound life changes, including medication burden, difficulty to retain their job and dependency on family members for self-care. The historical context emerged through older participants’ accounts of shifting societal perceptions of diabetes from a stigmatized condition to a manageable chronic disease. While some participants, particularly those with recent diagnoses or good glycemic control, reported minimal life changes beyond medication adherence, others described extensive lifestyle modifications affecting their daily routines, emotional well-being, and professional lives.

“So, because of all these medical conditions, my company told me that I’m not fit to stand and work. They asked me to find maybe the lesser or find a better job, because aviation is always long-standing work. Everywhere in the airport is a busy place. They say, “Due to your condition level, health issues, we don’t want you to suffer, make you suffer in this kind of line.” So, they asked me to leave the force.” (47 years, female)

##### 3.3.1.3 Motivators for diabetes management

Personal factors that motivated diabetes management included desire to prevent complications, track treatment progress, maintain healthy glucose levels, control the disease, self-motivation, feel good, and become healthy. They also included past experience of complications or witnessing someone living with diabetes. Over a quarter were motivated by fear of diabetes-related complications, stemming from their knowledge, or seeing others suffer. They acknowledged costs associated with treating complications. This fear motivated better diabetes management. Fear of family members bearing consequences was also a motivator. Nearly half cited constant reminders and support from family and friends as motivators.

“The thing is, if I never motivate myself, I cannot move on in my life. I’m a person, that’s why I say I never depend on others. Very long time, ever since young, I only depend on myself. I motivate myself.” (47 years, female)

Overall, the narratives revealed diverse motivational factors for diabetes management across different patient groups. Personal motivators varied by age and disease experience - younger participants were primarily motivated by immediate health goals and professional considerations, while older participants and those with complications were driven by fear of deterioration and previous negative health experiences. Support systems showed different patterns across care settings: ICC patients highlighted the motivational impact of structured feedback systems (e.g. CGMs) and healthcare team support, while primary care patients emphasized family support and peer groups. Notably, the presence of complications emerged as a strong motivator across all groups, though the response to this motivation manifested differently - some focused on preventing further complications while others sought to maintain independence. The narratives also suggested that younger participants particularly valued peer support groups and technological feedback mechanisms, while older participants relied more heavily on family support and healthcare team for motivation.

##### 3.3.1.4 Current self-care behaviors

This code captured current self-care behaviors participants undertook to manage diabetes. These included using CGM, exercising, following personalized diets, watching their diet (eating healthy, reducing carbohydrates and sugary drinks), reducing dining out, eating home-cooked food, eating before 7 pm, taking medications regularly, performing foot care, maintaining healthy sleep patterns, and managing weight. Overall, active self-care behaviors were noted, mainly diet- and physical activity-related changes. Only a few performed regular home glucose monitoring and foot care. Age-related differences were evident in self-care approaches: younger participants demonstrated greater adoption of technological tools for lifestyle management and structured exercise routines, while older participants emphasized traditional dietary modifications and medication adherence. Physical activity among older participants was predominantly limited to walking, largely due to diabetes-related complications and comorbidities. Notably, self-care behaviors including regular foot care and systematic glucose monitoring were more common among those with high SES, complications or those receiving specialized care, suggesting that care setting, socioeconomic status and disease severity influenced the breadth and depth of self-management practices.

“So I have a rice cooker that cooks rice like that, and then it leaves the rice grains up above the fluid, and then it re-sinks the rice grain to rehydrate it with, uh, water. So it tastes like rice but it is almost empty of, uh, sugar.” (47 years, male)

##### 3.3.1.5 Alternative therapies

Less than half of the participants had tried alternative therapies for diabetes, which included herbal medicine or ayurveda. However, participants did this along with their routine diabetes care as a supplementary medicine rather than a replacement therapy. The common herbal medicines used were moringa leaves, Lady's finger, fennel seed, cumin seeds, cinnamon and ginger, and bitter gourd. This pattern of complementary medicine use appeared consistent across different care settings and age groups, suggesting that cultural beliefs about traditional remedies coexisted with, rather than competed against, modern medical approaches to diabetes management.

“Last time I would drink the bitter gourd juice. Actually, but once I drink that, it really drops very low because I was eating the medicine as well as the bitter gourd juice. They tell me that actually it’s a wrong system. They told me that I should drink the bitter gourd juice. For me, I’m very greedy because I want my sugar level to go down. Every day, empty stomach, I’ll drink the bitter gourd juice.” (47 years, female)

### 3.4 Met and unmet needs

**Figure 2:**
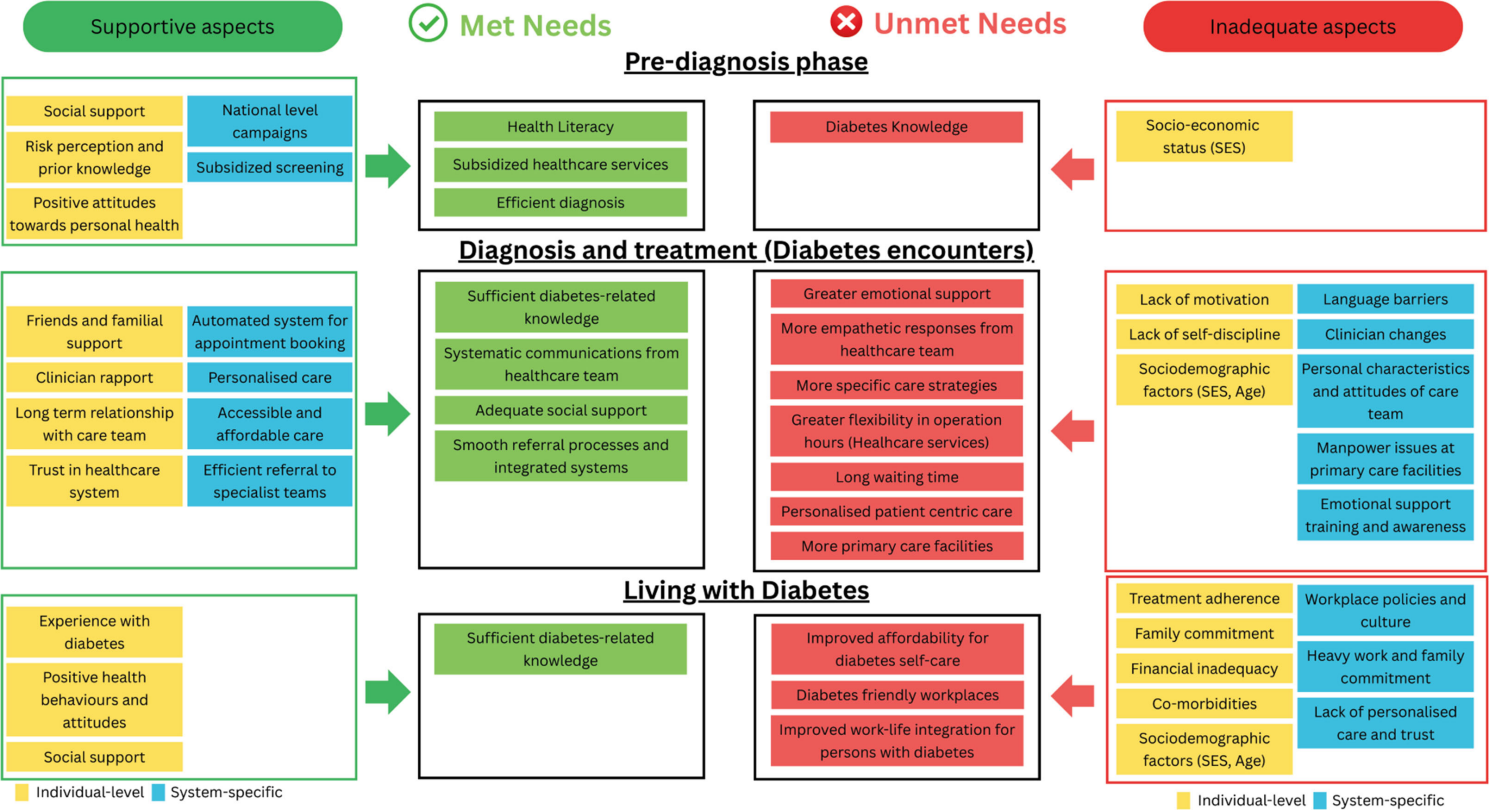
Conceptual map showing the interplay between personal and system-level factors and their influence on patients’ met and unmet needs.

A conceptual map of met and unmet needs encountered in various stages and their potential interaction with system level and individual factors have been populated in **Figure 2**. The overall unmet needs included the need for a personalized and patient-centric approach.

## 4 Discussion

The current study explored experiences of patients as they navigated through Singapore’s healthcare system. This is the first study to examine a detailed experience map of the journey of diabetes patients identifying avenues for healthcare system improvement. Three main stages of the journey were noted: pre-diagnosis (stage 1), diagnosis and treatment (stage 2), and living with diabetes (stage 3). Stage 1 included themes of awareness and choice of care provider for initial care and showed few care gaps. Stage 2 identified service gaps and unmet needs. The themes that emerged included acceptance and facing life with diabetes, and diabetes disease encounters, with several codes captured under each theme. Stage 3 included themes related to diabetes self-care. While the pre-diagnosis phase that included symptom awareness and initial healthcare contact, was seamless, challenges and improvement areas were identified in the treatment and management phase. Participants recognized healthcare system efficacy but had mixed responses toward experiences with healthcare teams across settings and referrals. Participants shared barriers and enablers of diabetes management. Several codes on diabetes self-care emerged, including narratives of motivators and challenges in self-care.

### 4.1 Pre-diagnosis experience

In the pre-diagnosis phase, a significant proportion of participants underwent regular health screening that were self-initiated, part of annual work screenings, or occurred during their treatment for existing conditions as noted by other studies (15). Participants generally had good diabetes awareness, gathering information from various sources as reported in previous studies (16). The anti-diabetes campaigns by the Singapore government and health Promotion Board, such as ‘War on Diabetes’ (7), along with the subsidized ‘Screen for Life’ program (subsidized screening for S$5 or below for those over 40 years old) (17), likely contributed to positive experiences and met needs observed in this domain. All participants accessed primary healthcare systems for initial diagnosis and care. Affordability, prior system contact, and clinician characteristics influenced care provider choice, similar to findings in other qualitative studies (15).

### 4.2 Treatment and follow-up challenges

Participants expressed challenges and areas for improvement in treatment and follow-up domains. Despite perceived healthcare system efficiency, patient management (long waiting times at the polyclinic, multiple external referrals, and brief consultations), and diabetes treatment costs were concerns. Long waits resulted in short consultations, hindering effective communication and patient dissatisfaction. Similar experiences were reported in other studies. Nocon et al. (18), compared 19 primary care clinics, finding similar patient experiences with a subset of the clinics had long waiting times, poor communication, and higher costs. A similar study in Scotland reported minimal duration of consultation, inconvenience, and frustrations with referrals outside the primary care setting (19). Ide et al. (20), noted that provider shortages, longer waiting times, minimal time with clinicians, and higher patient loads were the system-level barriers to diabetes care. Rushforth et al. (21), shared similar perspective where clinicians expressed frustration with the demands of diabetes care in the context of limited resources and time, resulting in a lack of confidence, anxiety, and frustration. These findings suggest an imperative need to expand the primary care network for diabetes patients. Participants also suggested expanding the healthcare workforce by increasing the number of clinicians or establishing additional polyclinics in strategic locations to manage the challenge.

In contrast to the primary care experience, those attending ICCs expressed satisfaction with the setting, services, and holistic care received. Other studies reported similar positive narratives about ICCs (22, 23), with a scoping review showing improved clinical outcomes, self-efficiency, and patient satisfaction. A mixed-methods study from Singapore reported positive experiences shared by the patients regarding their diabetes care in the primary care setting, contrary to the findings of the current study (24). The patients in the study endorsed receiving appropriate support, patient-centric care, continuity of care, convenient access, and affordability. This discrepancy could be explained as the study represented GP clinics and the care team was involved in the in-depth interviews, while in the current study, experienced interviewers from the collaborating institutions who were not involved in the care team conducted the interviews that mitigates response bias. Both studies, however, shared common themes as well such as the preference for seeing the same doctor, concerns regarding cost, preference for ICCs (24). Nonetheless, the participants in the current study were users or had sought care at ICC while those from Goh et al. (24), had not experienced the ICCs. These findings suggest a need for more affordable, high-quality ICCs for diabetes care.

### 4.3 Age-specific challenges

The current study revealed differing perspectives and challenges for younger participants with early-onset diabetes, including difficulty accepting diagnosis and self-management challenges. Current diabetes care does not differentiate between age groups who have different care needs. Studies show rising diabetes rates among young adults, with obesity, family history, and lifestyle as major risk factors (25). Young-onset diabetes has an aggressive phenotype with multiple complications and adverse long-term outcomes (26). Smith et al. (27), found younger-onset diabetes patients had poor self-management, higher smoking rates, complications, depressive symptoms, and more emergency department visits. Evidence suggests diabetes care should be tailored to different age groups. For younger patients, psychological support post-diagnosis and peer support groups could alleviate distress. Clinicians should spend more time with younger patients due to their different demands and expectations, as unmet needs may lead to non-adherence, poor self-care, increased morbidity, and adverse long-term outcomes.

### 4.4 Enablers and barriers to treatment

The enablers of the treatment included the healthcare system (shorter waiting time, accessibility, ambience, patient management), and team-related factors (positive and welcoming attitudes of the healthcare team, personalized, and patient-centered care). Patients, regardless of the setting, were happy with the overall efficiency of the healthcare platforms, which included the ease of appointment booking, the apps for self-management of appointment and visit-related information, the reminders, etc. However, less than a quarter of the primary care experiences included narratives of empathetic, respectful, and understanding team members. Similar narratives were reported in other qualitative studies as well (28). Similar to the current study, other studies also reported satisfaction with communication and collaborating care between primary care clinicians and specialists during referrals (24). A systematic review of mixed-methods studies showed that the patients also valued caregiver-patient communication as a cornerstone of the treatment journey that could promote adherence to treatment, self-management, self-efficiency, quality of life, and reduce diabetes-related distress (29). Given the importance of communication in improving treatment adherence and self-care behaviors, healthcare systems focus on enhancing patient-carer interactions by allowing more engagement time.

### 4.5 Diabetes self-management

Participants highlighted motivators for self-management including preventing complications, tracking treatment progress, maintaining healthy blood sugar levels, controlling the disease, feeling good, and becoming healthy. Past experiences with complications, witnessing others suffer, and family/friend influence were major motivators, which aligns with findings from other studies (30, 31). Personal responsibility was the main facilitator, prompting self-care adoption to prevent complications, which is fundamental to diabetes self-care (32). Barriers to self-care included difficulty giving up tasty food and other commitments. Bukhsh et al. (30), reported similar challenges in diet management in social settings. Other barriers included higher costs of healthier options, cravings for sugary foods, medication forgetfulness, and hectic work schedules. Participants also adopted alternative treatments like herbal or ayurvedic approaches, influenced by internet and family/friends, similar to findings by Adhikari and colleagues (31).

### 4.6 Recommended improvements

The care delivery could be improved by incorporating several operational reforms. These include enhanced digital platforms that allow patients to check doctor availability and choose their preferred physicians. A care continuity model in primary care, similar to the ICC, would be beneficial, where clinicians and nurses work in pairs to manage patients. Additionally, developing multilingual education programs, peer support networks, and mental health support within the clinic would enhance patient care. Given the diversity of the patient population, implementing risk stratification for fast-tracked appointments for those in need, improving existing resource distribution, and introducing group consultations would improve patient satisfaction and care outcomes. Currently, there are no specific diabetes-related services for the younger age group. This age group, because of their unique challenges, would benefit from age-appropriate support systems such as peer-mentoring, younger age group-oriented clinics, support groups, extended consultations, or digital health platforms. Mental health support is also a critical component to enable them to cope with the diagnosis and support them during their journey. These steps could bridge the gap in care delivery for this specific age group.

### 4.7 Strengths and limitations of the study

The study has several strengths and limitations. The strength includes examination of multiple stages of diabetes care, inclusion of different demographic groups and care settings, and multilingual interviews that increased the sample variation and data richness. The limitation is that the participants from the polyclinics and integrated healthcare systems and the ones who came through referral were recruited in the study with none currently seeking treatment with GPs. Limitations also include the absence of participants from other healthcare providers, and potential temporal discrepancies due to ongoing healthcare system transformations. Private healthcare organizations manage a substantial proportion of individuals with diabetes, and the underrepresentation of their experiences may skew the data and limit the generalizability of the findings. Additionally, the study did not include participants from private hospitals who might have a different experience. Future studies should include different healthcare settings to capture a wider perspective regarding diabetes care, which allows comparison of care across different healthcare systems. Such journeys will enable the healthcare system to gain knowledge from successful journeys and disengagement from healthcare services to get a better understanding of contributing factors to making system-level changes. The healthcare system is undergoing significant transformation with the implementation of Healthier SG and polyclinic capacity expansion. The current study may not have captured this evolving context. Additionally, the multiple triangulation approach is a major strength that support the validity of the findings. Also, the study was conducted in a high-income urban setting, which limits its transferability to low- and middle-income settings.

### 4.8 Broader implications

Although this study represents Singapore’s healthcare context, the results demonstrate broader applicability across diverse healthcare systems. Main findings regarding the need for patient-centered care delivery, therapeutic relationships, age-specific interventions, and barriers and facilitators for diabetes management represent universal challenges in chronic disease management. While certain operational elements, such as systematic specialist care referral pathways and subsidized screening programs, are specific to Singapore’s healthcare framework, the observations regarding the influence of cultural diversity on diabetes management are relevant to other multicultural urban healthcare settings.

## 5 Conclusion

The study delineated diabetes patients’ experiences across three phases: pre-diagnosis, diagnosis/treatment, and self-management. The pre-diagnosis phase was relatively uncomplicated with participants expressing sufficient knowledge, no unmet needs and positive help- seeking behaviors. However, significant challenges emerged in the treatment and self-management domains, particularly at the healthcare system and team levels. The current primary care system exhibits some shortcomings in tailoring care for younger people with diabetes who have distinct needs. Primary care networks, despite robust infrastructure, are characterized by extended waiting periods, resulting in perfunctory interactions and patient dissatisfaction. In contrast, participants attending the ICC, regardless of their SES status, age, and duration of diabetes, expressed satisfaction with their care experiences. This satisfaction stemmed from operational efficiency (including ease of appointment booking, shorter waiting times, amenities, joint consultations, and integrated care facilities) and the empathetic, friendly, and caring attitude of the healthcare team. Patients expressed a strong preference for personalized, patient-centric care. While patient complexity (including medication regimen, complications, and socioeconomic status) did not influence care experiences, the healthcare setting and provider attitudes emerged as critical determinants of patient satisfaction, with ICC demonstrating superior care experiences compared to primary care settings. These findings have implications for developing novel approaches to address identified concerns and manage patients with heterogeneous needs.

## Data Availability

The original contributions presented in the study are included in the article/**Supplementary Material**. Further inquiries can be directed to the corresponding author.
