# Supplementary material for: Through the eyes of the patients: a qualitative study of diabetes patients’ experiences navigating the healthcare system
Source: Front Endocrinol (Lausanne). 2025 Aug 26;16:1588192. doi: 10.3389/fendo.2025.1588192 (PMC12417109; doi:10.3389/fendo.2025.1588192)
Supplement: Supplementary file 1 [file Table1.docx]

Supplementary Material

# Supplementary Table S1: Consolidated criteria for reporting qualitative studies (COREQ): 32-item checklist

| **No** | | **Item** | **Description** | **Section #** | |
| --- | --- | --- | --- | --- | --- |
| **Domain 1: Research team and reflexivity** | | | | | |
| **Personal characteristics** | | | | | |
| *1.* | | Interviewer/facilitator | Which author/s conducted the interview or focus group? | Procedure | |
| *2.* | | Credentials | What were the researcher's credentials? *E.g. PhD, MD* | Supplementary Table S2 | |
| *3.* | | Occupation | What was their occupation at the time of the study? | Supplementary Table S2 | |
| *4.* | | Gender | Was the researcher male or female? | Supplementary Table S2 | |
| *5.* | | Experience and training | What experience or training did the researcher have? | Supplementary Table S2 | |
| Relationship with participants | | | | | |
| *6.* | | Relationship established | Was a relationship established prior to study commencement? | Not applicable | |
| *7.* | | Participant knowledge of the interviewer | What did the participants know about the researcher? *E.g. Personal goals, reasons for doing the research* | Procedure | |
| *8.* | | Interviewer characteristics | What characteristics were reported about the interviewer/facilitator? *E.g. Bias, assumptions, reasons and interests in the research topic* | Reflexivity & Supplementary Table S2 | |
| **Domain 2: Study design** | | | | | |
| **Theoretical framework** | | | | | |
| *9.* | | Methodological orientation and theory | What methodological orientation was stated to underpin the study? *E.g. grounded theory, discourse analysis, ethnography, phenomenology, content analysis* | Design | |
| **Participant selection** | | | | | |
| *10.* | | Sampling | How were participants selected? *E.g. purposive, convenience, consecutive, snowball* | Sampling and sample size | |
| *11.* | | Method of approach | How were participants approached? *E.g. face- to-face, telephone, mail, email* | Participants | |
| *12.* | | Sample size | How many participants were in the study? | Sampling and sample size  Results & Table 1 | |
| *13.* | | Non-participation | How many people refused to participate or dropped out? What were the reasons for this? | Results | |
| **Setting** | | | | | |
| *14.* | | Setting of data collection | | Where was the data collected? *E.g. home, clinic, workplace* | Data collection |
| *15.* | | Presence of non- participants | | Was anyone else present besides the participants and researchers? | Reflexivity |
| *16.* | | Description of sample | | What are the important characteristics of the sample? *E.g. demographic data, date* | Results & Table 1 |
| **Data collection** | | | | | |
| *17.* | | Interview guide | | Were questions, prompts, guides provided by the authors? Was it pilot tested? | Supplementary  Table S3 |
| *18.* | | Repeat interviews | | Were repeat interviews carried out? If yes, how many? | Data collection |
| *19.* | | Audio/visual recording | | Did the research use audio or visual recording to collect the data? | Data collection |
| *20.* | | Field notes | | Were field notes made during and/or after the interview or focus group? | Reflexivity |
| *21.* | | Duration | | What was the duration of the interviews or focus group? | Data collection |
| *22.* | | Data saturation | | Was data saturation discussed? | Sampling and sample size |
| *23.* | | Transcripts returned | | Were transcripts returned to participants for comment and/or correction? | Not applicable |
| **Domain 3: analysis and findings** | | | | | |
| **Data analysis** | | | | | |
| *24.* | | Number of data coders | | How many data coders coded the data? | Analysis |
| *25.* | | Description of the coding tree | | Did authors provide a description of the coding tree? | Analysis |
| *26.* | | Derivation of themes | | Were themes identified in advance or derived from the data? | Analysis |
| *27.* | | Software | | What software, if applicable, was used to manage the data? | Analysis |
| *28.* | | Participant checking | | Did participants provide feedback on the findings? | Not applicable |
| **Reporting** | | | | | |
| *29.* | | Quotations presented | | Were participant quotations presented to illustrate the themes / findings? Was each quotation identified? *E.g. Participant number* | Results |
| *30.* | | Data and findings consistent | | Was there consistency between the data presented and the findings? | Results |
| *31.* | | Clarity of major themes | | Were major themes clearly presented in the findings? | Results & Figure 1 |
| *32.* | | Clarity of minor themes | | Is there a description of diverse cases or discussion of minor themes? | Results and Figure 1 |

# Supplementary Table S2: Characteristics of team members

| **Team member** | **Initials** | **Credentials** | **Occupation** | **Gender** | **Training** | **Study role** |
| --- | --- | --- | --- | --- | --- | --- |
| PV AshaRani | AR | Ph.D | Senior Manager Research | female | Mental Health, Qualitative Research, Health Service Research | Conceptualisation, preparation of interview guide, develop study methodology, ethics process, recruitment, conducting interviews, note taking during interviews, debriefing, quality control of transcripts, coding, discussion, thematic analysis, preparation of the first draft of the manuscript. |
| P. Kumarasan Roystonn | KR | M.Sc | Research Officer | male | Psychology | Recruitment, conducting interviews, note taking during interviews, proofreading transcripts, data curation, quality control of transcripts, coding, discussing codes, thematic analysis, manuscript preparation (review & editing) |
| Madhumitha Ramu | MR | BSocSc | Research Assistant | female | Psychology, Qualitative Research | Recruitment, conducting interviews, note taking during interviews, proofreading transcripts, data curation, quality control of transcripts, coding, discussing codes, thematic analysis, manuscript preparation (review & editing) |
| Yeow Wee Brian Tan | BT | BPsychSc (Hons) | Research Officer | male | Public Health, Psychology, Quantitative Research, Qualitative Research | Recruitment, conducting interviews, note taking during interviews, proofreading transcripts, data curation, quality control of transcripts, coding, discussing codes, thematic analysis, manuscript preparation (review & editing) |
| Fiona Devi | FD | BA | Research Officer | female | Qualitative Research, Mental Health | Recruitment, conducting interviews, note taking during interviews, proofreading transcripts, data curation, quality control of transcripts, coding, discussing codes, manuscript preparation (review & editing) |
| Muhd Iskandar Shah | IS | BA | Research Officer | male | Psychology | Recruitment, conducting interviews, note-taking interviews, proofreading transcripts, coding, discussing codes, manuscript preparation (review & editing) |
| Wang Peizhi | WP | MPH | Research Psychology | female | Psychology, Public Health | Recruitment, Interviews, note-taking during interviews, proofreading transcripts, coding, discussing codes, manuscript preparation (review & editing) |
| Sum Chee Fang | SCF | MBBS,  FRCPE | Senior Consultant Endocrinologist | male | Internal Medicine  Endocrinology | Conceptualisation, contribute to the preparation of interview guide and study methodology, assist with recruitment, manuscript preparation (review & editing) |
| Tavintharan Subramaniam | TS | MBBS, MRCP | Senior Consultant, Endocrinologist | male | Physician, Diabetologist, Qualitative And Quantative Research | Methodological advice, assist with recruitment, manuscript preparation (review & editing) |
| Lee Eng Sing | LES | Ph.D | Medical Director | male | Family Medicine, Qualitative & Quantitative Research | Methodological advice, assist with recruitment,manuscript preparation (review & editing). |
| Chong Siow Ann | CSA | MD | Senior Consultant | male | Psychiatry, Qualitative Research, Epidemiology, Public Health | Conceptualisation, methodological advice, manuscript preparation (review & editing) |
| Mythily Subramaniam | MS | Ph.D | Assistant Chairman  Medical Board | female | Mental Health, Epidemiology, Psychology, Quantitative Research, Qualitative Research | Conceptualisation, preparation of interview guide, develop study methodology,interviews, coding and code discussion, thematic analysis, manuscript preparation (review & editing) |

# Supplementary Table S3: Interview Guide for Patient Journey for Individuals with Type 2 Diabetes

| **Initial Question** | **Probes** |
| --- | --- |
| **Introduction** | |
| Can you tell me about when did you first learn that you had diabetes? | 1. Can you tell me more about the time of diagnosis? 2. How did you realise that you had diabetes? 3. Can you tell me more about the healthcare setting you were diagnosed? |
| What is your current experience of living with and managing diabetes? |  |
| **Barriers and facilitators in navigating care and services** | |
| **Refer to the sociodemographic section(Which healthcare setting(s) are you currently seeking care for diabetes) and chose the respective questions for specific healthcare provider** | |
| *If GP/polyclinic* |  |
| You said that you usually follow up with your GP/ polyclinic doctor. Why is that so? |  |
| Can you share what happens during a typical visit with your GP/ polyclinic doctor? |  |
| What are some things that you like about these visits? | Why? |
| What are some things that you do not like about these visits? | Why? |
| Do your GP/ polyclinic doctor refer you to ancillary services such as such as diabetic nurse counselling, diabetic eye screening, diabetic foot screening, annual checks on your kidneys etc. for your diabetes care? | If so, what are these services? such as diabetic nurse counselling, diabetic eye screening, diabetic foot screening, annual checks on your kidneys etc.   1. Can you describe how these services are delivered to you? 2. Do you have to make an appointment yourself or does the doctor do it for you? 3. How easy or difficult was it for you? 4. Which part was easy, and which was difficult? 5. How often do you go for such ancillary services, and the reasons for doing/not doing so? |
| - *If the response is specialist/ endocrinologist in hospitals ask:* |  |
| You said that you usually follow up with your specialist/ endocrinologist. Why is that so? |  |
| How often do you follow up with your specialist/ endocrinologist? | 1. How would you describe the referral process and the waiting times to get to see your specialist? 2. Are you still following up with your primary care doctor? If yes probe for more information. |
| Can you share what happens during a typical visit with your specialist/ endocrinologist? |  |
| - What are some things that you like about these visits? | Why? |
| What are some things that you do not like about these visits? | Why? |
| Do your specialist/ endocrinologist refer you to ancillary services for your diabetes care? | If so, can you tell me more about these services?   - - 1. Can you describe how these services are delivered to you?     2. How easy or difficult was it for you?     3. Which part was easy, and which was difficult?     4. How often do you go for such ancillary services, and the reasons for doing/not doing so? |
| - *If the response include other healthcare professionals e.g. podiatrist, dietitian/ nutritionist ask:* |  |
| You said that you follow up with your podiatrist, dietitian/ nutritionist. Why is that so? |  |
| Can you share what happens during a typical visit with your podiatrist, dietitian/ nutritionist? |  |
| What are some things that you like about these visits? |  |
| What are some things that you do not like about these visits. | Why? |
| What do you have to do to receive these services? |  |
| How easy was it for you? | Which part was easy? Can you tell me more? |
| How difficult was it for you? | Which was difficult? Can you tell me more? |
| Do you follow up on the referrals made by your main healthcare professional? | Why or Why Not? |
| How do you manage all these (different) appointments? |  |
| What are some of the challenges you have faced in following up with multiple service providers? |  |
| What are some of the things that have helped you in this process? |  |
| Are there any changes/areas of improvement to make it easier for you or your caregivers to attend these appointments? |  |
| Do you think that the healthcare professionals e.g. nurses, doctors, clinic staff/ hospital staff , including those from the community involved in your diabetes care communicate with each other about your condition and its treatment? |  |
| Can you tell me about an instance when you felt they did not communicate well? | How did this affect you? |
| What do you think is most important in a patient–carer (healthcare professional) relationship that helps you manage your diabetes well? |  |
| Have your healthcare professional worked with you to set goals in managing your condition? | 1. Can share how was this done? 2. How often do you have such goal setting discussions? |
| Do you think this has been beneficial in improving your diabetes management? | Why or why not? |
| Has it happened that you are in need of medical information but were unsure where to seek help? | Could you please give examples of how you solved it?  Can you tell me where you received information? |
| May I know if you have ever sought treatment at the A&E for your diabetes care? | 1. Can you tell me what happened? 2. What did you think of the experience? |
| May I know if you have ever been admitted to any hospital for your diabetes care? | 1. Can you tell me what happened? 2. What did you think of the experience? |
| Can you tell me if I have missed out on any other service provider/ professional/ any other person who helps you with your diabetes management? | If yes – Can you tell me how this person helps you? |
| **Barriers and facilitators to management and control** |  |
| How would you describe your current control of diabetes? |  |
| May I know what are some of the things you do to manage your diabetes | For example taking your medicines regularly, blood sugar monitoring, foot care, diet, regular exercise, etc |
| Has anything or anyone helped you to manage your diabetes well (for example sources of info, peer support group, health peers, lifestyle management sessions, family support, apps etc.)? |  |
| What are some of the challenges you face in self-management? |  |
| How do you motivate yourself to take good care of yourself? |  |
| What are some of the factors you think will enable those with diabetes to lead a healthy lifestyle? |  |
| If everything was possible, do you have any thoughts about how diabetes care could be improved to make it easier for you to live with diabetes and control it? |  |
| **END** | |
